# Determining the Environmental Factors Underlying the Spatial Variability of Insect Appearance Phenology for the Honey Bee, *Apis mellifera*, and the Small White, *Pieris rapae*

**DOI:** 10.1673/031.010.3401

**Published:** 2010-04-15

**Authors:** Oscar Gordo, Juan José Sanz, Jorge M. Lobo

**Affiliations:** ^1^ Departamento de Ecología Evolutiva, Museo Nacional de Ciencias Naturales — CSIC, Madrid, Spain.; ^2^ Departamento de Biodiversidad y Biología Evolutiva, Museo Nacional de Ciencias Naturales — CSIC, Madrid, Spain

**Keywords:** appearance date, climate, geographic information system, Iberian peninsula, land use, modelling, normalized difference vegetation index, topography

## Abstract

The spatial patterns of the variability of the appearance dates of the honey *bee Apis mellifera* L. (Hymenoptera: Apidea) and the small white *Pieris rapae* (L.) (Lepidoptera: Pieridae) were investigated in Spain. A database of more than 7,000 records of the dates of the first spring sightings of each species in more than 700 localities from 1952–2004 was used. Phenological data were related to spatial, topographical, climate, land use, and vegetation productivity explanatory variables by means of multiple regression models in order to search for the environmental mechanisms underlying the observable phenological variability. Temperature and altitudinal spatial gradients accounted for most of the spatial variability in the phenology of the studied species, while vegetation productivity and land use had low relevance. In both species, the first individuals were recorded at those sites with warmer springs and dry summers, at low altitudes, and not covered with dry farming (i.e., cereal crops). The identity and magnitude of the effect of the variables were almost identical for both species and closely mirrored spatial temperature gradients. The best explanatory models accounted for up to half of the variability of appearance dates. Residuals did not show a spatial autocorrelation, meaning that no other spatially structured variable at our working resolution could have improved the results. Differences in the spatial patterns of phenology with regard to other taxa, such as arrival dates of migratory birds, suggest that spatial constraints may play an essential role in the phenological matching between trophic levels.

## Introduction

Phenology is the seasonal timing of life history events of organisms. Proper timing of life events — such as emergence, dispersal and reproduction — is a keystone for individual survival and success. Therefore, the phenology must evolve to match the environment in a manner that optimizes fitness, and traits that determine timing are presumably under strong selection (Wiklund and Fagerström 1977; Iwasa and Levin 1995; Jonzén et al. 2007). Evidence of the finetuning between phenology and environment would be the rapid alteration detected in the life cycles of a wide array of species in response to recent climate change (Parmesan and Yohe 2003; Root et al. 2003, 2005).

In contrast to the significant attention recently paid to the temporal changes in phenological traits, the spatial variability of phenology remains poorly studied. More knowledge is needed regarding both the description of spatial variation patterns of phenology and also the search for potential environmental, biological or evolutionary mechanisms shaping them. The asymmetry between the temporal and spatial focus of current phenological research may constrain the abilities to fully understand factors controlling phenology and to make accurate predictions about the impact of climate change on organisms' phenology. Therefore, a better knowledge of factors governing the spatial variability of phenology is essential for fully understanding how and why phenology is changing with time.

To date, insect macroecology has focused mainly on large-scale patterns of distribution (Eyre et al. 2003, 2005a; Jiménez-Valverde et al. 2007) or diversity (Hawkins and Porter 2003; Stefanescu et al. 2004; Baselga and Jiménez-Valverde 2007) of insect species. Meanwhile, large-scale patterns of other biological features of insects, such as their phenology and its causes, remain unexplored. Data requirements are one possible reason for the lack of research in this area. In fact, reliable results and sound conclusions can only be derived from huge datasets gathered by many people involved in perfectly coordinated sampling networks. Such collaborative efforts in Britain enabled the use of 1.5 million records collected between 1995 and 1999 (Asher et al. 2001) in the exploration of spatial trends in the mean sighting dates of 29 resident butterfly species (Roy and Asher 2003). The sighting dates showed marked latitudinal and longitudinal gradients through Britain for most species. However, some butterfly species did not show spatial patterns in their phenology. This result is striking because it is expected that the spatial variability of insect phenology should mirror gradients of the most influential environmental variables affecting the timing of insect lifecycles, such as temperature (e.g., Fielding et al. 1999). These species lacking obvious spatial patterns may require stronger environmental gradients than those gradients that occur in Britain in order to offer a measurable gradient of phenology. Alternatively, there may be other physiological or behavioural mechanisms for ensuring a macroscale synchronous phenology that could be more beneficial than regional differences in phenology. In any case, these results stress the necessity of more studies on spatial patterns of insect phenology and on the mechanisms that influence them (Weiss et al. 1988, 1993; Fielding et al. 1999).

Another example of the value of monitoring networks for the assessment of the spatial variability of phenology is offered by recent studies based on the EXAMINE project (Cocu et al. 2005; Harrington et al. 2007). The capture of flying aphids in a network of suction traps spread over Western Europe allowed the determination of the geographical and environmental factors related to the spatial variability of both the numbers and the phenology of some aphid species. Regarding phenology, these studies demonstrated that differences in flying periods among trapping sites were due to differences in climatic conditions prevailing in them. This result was expected due to the well-known effect of climate over aphid activity (Worner et al. 1995; Zhou et al. 1995, 1996). However, spatial trends of aphid phenology through Europe were also explained by other environmental features, such as land use in the area surrounding each trap (Cocu et al. 2005; Harrington et al. 2007). Moreover, the effect of climate, land use or geography on aphid phenology varied according to the working scale (Cocu et al. 2005). This research on aphid phenology both provided insights into the complexities involved in spatial aspect phenology and also stressed the urgent necessity to devote more efforts toward this largely unexplored issue.

In a previous study, Gordo and Sanz (2006b) demonstrated that the honey bee, *Apis mellifera* (L.) (Hymenoptera: Apidea), and small white, *Pieris rapae* (L.) (Lepidoptera: Pieridae), phenologies had significant latitudinal, longitudinal and altitudinal trends in the Iberian Peninsula. Unfortunately, variables such as latitude or longitude do not provide conclusions concerning the possible environmental or biological factors underlying the observable differences in the appearance schedule of the insects among study sites. The aim of Gordo and Sanz (2006b) was to determine the temporal aspect of phenology, and the spatial variables were used as covariates in order to account for the possible confounding effects of spatial gradients on temporal trends across the Iberian Peninsula. Nonetheless, Gordo and Sanz (2006b) suggest the existence of a spatially structured variability in the appearance dates of *A. mellifera* and *P. rapae.* Therefore, these species have become ideal candidates for a comprehensive study about the spatial variability of insect phenology. The aim of this study is to complete the temporal picture explored by Gordo and Sanz (2006b) by further investigating the spatial patterns of the appearance dates of *A. mellifera* and *P. rapae* in Spain, together with an exhaustive search for the underlying environmental factors. Therefore, the present study is focused on the patterns and causes of phenological variability among localities.

## Materials and Methods

### Phenological data

Since the 1950s, the first appearance dates for *A. mellifera* and *P. rapae* have been recorded by volunteer observers in hundreds of localities throughout Spain. These observers applied the standardized protocolsproposed by the Spanish *Intituto Nacional de Meteorología* (for more details about this scheme, see Gordo and Sanz (2006a, b)). Both species are widespread in Spain and are well-studied due to their relevance in agriculture. *A. mellifera* is the main pollinator for orchards and most fruit trees, while the larval stages of *P. rapae* are important pests of cabbage crops. Adults of *P. rapae* also may act as pollinators of some entomophilous crops.

**Figure 1.**
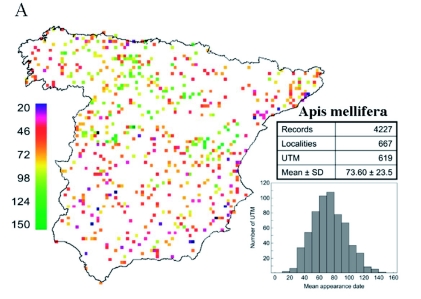
Maps of the distribution of phenological records of the appearance dates of *Apis mellifera.* Each square represents a UTM with phenological records, and its colour depicts the mean date. Scale color bar in Julian days (1 = 1 January). The total number of records, localities, and UTM together with the mean value and the standard deviation (SD) of all records are also specified. A histogram with the distribution of mean dates per UTM is also shown (scale of x-axis in Julian days). High quality figures are available online.

Records dated between 1952 and 2004 from original files of the *Intituto Nacional de Meteorología* were collected and digitized. A total of 7,263 records from 737 localities were gathered (see Figure 1 for details for each species). Each date was transformed into a Julian day scale (1 = 1 January). For leap years, one day was added after February 28 to take the extra day into account. Before performing any analyses, temporal trends of phenological data were removed by the regression of appearance dates against the quadratic function of the year for each species (Gordo and Sanz 2006b). Residuals obtained from temporal regression models were used as a measure of the phenological variation independent of the year from which these dates were recorded (see also Gordo et al. 2007a, b). Temporally corrected dates were used thereafter.

The mean appearance date for all records from the same 100 km^2^ (10 × 10 km) universal transverse mercator (UTM) cell was calculated for each species. Because some localities of the phenological network were in the same UTM cell, the final sample size available for calculations (i.e. different UTM cells) was smaller than the number of original localities (see Figure 1). Mean values for each UTM cell could be biased due to differences in the number of records. This possible dependence was tested by calculating Spearman rank correlations between mean values and number of records in each UTM cell (*A. mellifera: r*s = 0.034, *t*617 = 0.845, *P* = 0.395; *P. rapae: r*s = -0.011, *t*440 = -0.231, *P* = 0.817). Because the mean appearance date was not dependent on the number of records, all UTM cells with available records for both species were used.

### Explanatory variables *A. mellifera*

A total of 47 explanatory variables were classified into five categories (spatial, topographical, climatic, vegetation productivity and land uses; see Table 1) and used to model the appearance dates of *A.* *mellifera* and *P. rapae* throughout Spain. Seven topographic variables were obtained from a digital elevation model (Clark Labs 2000) for each of the 100 km^2^ UTM Iberian squares (n = 6063) using the IDRISI 32 Geographic Information System (Clark Labs 2001a). The mean, minimum and maximum altitude of each 100 km^2^ UTM cell was calculated from all 1 km^2^ pixels included in each 100 km^2^ UTM cell. The altitude range, slope, aspect (the mean direction of the slope) and diversity of aspects were also obtained for each 100 km^2^ UTM cell. Delayed appearance dates are expected in UTMs at higher elevations and/or with northern exposures (Scott and Epstein 1987; Weiss et al. 1988, 1993; Gutiérrez and Menéndez 1998; Cocu et al. 2005; Gordo and Sanz 2006b; Harrington et al. 2007).

Eighteen climatic variables were provided by the *Instituto Nacional de Meteorología* for each of the 100 km^2^ UTM Iberian squares. Climatic variables were rainfall and minimum, mean, and maximum temperatures during each season (spring, summer, autumn and winter), together with the annual temperature range and an aridity index. Spring was defined as April, May and June, summer as July, August and September, autumn as October, November and December, and winter as January, February and March for all seasonal variables. The aridity index was calculated as: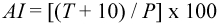 where *P* is the annual precipitation, and *T* is the mean annual temperature. Later appearance dates were expected in UTMs with cooler temperatures due to the strong effect of this variable on the development and activity of insects (Scott and Epstein 1987; Zhou et al. 1995; Sparks and Yates 1997; Fielding et al. 1999; Dell et al. 2005; Gordo and Sanz 2006b). In the case of precipitation, later appearance of insects was predicted for the UTMs that were more moist, especially during the spring (Cocu et al. 2005; Harrington et al. 2007). Therefore, the more arid areas (i.e., higher AI values) should be also those with the earliest appearance of bees and butterflies.

Vegetation productivity also was evaluated as a possible explanatory variable for spatial patterns of the spring appearances of *A. mellifera* and *P. rapae* in the Iberian Peninsula. This variable was measured as the normalized vegetation difference index (NDVI). The NDVI is the normalized difference between red (0.55 – 0.68 µm) and infrared (0.73 – 1.1 µm) reflectance, as measured by the National Oceanic and Atmospheric Administration's polar orbiting satellite's advanced very high resolution radiometer sensor (Smith et al. 1997). The NDVI is determined by the degree of red wavelength absorption by chlorophyll, which is proportional to leaf chlorophyll density, as well as by the reflectance of near infrared radiation, which is proportional to green leaf density (Tucker et al. 1985). Therefore, the NDVI correlates well with variables such as green leaf biomass, leaf area index, total accumulated dry matter and annual net primary productivity (Nicholson et al. 1990). NDVI data were available from Clark Labs world images as monthly values from 1982 to 2000 at a spatial resolution of 0.1 degree (Clark Labs 2001b). A value of vegetation productivity for each 100 km^2^ UTM of the Iberian Peninsula was calculated by averaging monthly images available for each season between 1982–2000. More productive areas were expected to support higher abundance and diversity of organisms of upper trophic levels, such as insects (Hawkins and Porter 2003; Bailey et al. 2004; Seto et al. 2004). Larger populations of insects may enhance an early appearance simply due to increased chances for detection of early individuals (Tryjanowski et al. 2005; Dennis et al. 2006). Alternatively, larger populations may indeed be related to an earlier appearance because of the greater genetic diversity and the consequent higher probabilities for early phenotypes.

Land use types were also included because features of the environment eventually determine the presence of insect species (Eyre et al. 2003, 2005b). Therefore, habitat availability can be considered as another surrogate for the abundance of populations at a local scale, which, in turn, can affect detection (see above). Furthermore, land use has been demonstrated to have effects on the phenology of other insect taxa, such as aphids (Cocu et al. 2005; Harrington et al. 2007). Consequently, it would be of interest to understand to what extent this evidence is applicable to different taxonomic groups, such as bees or butterflies. The distribution of 15 land use types for the Iberian Peninsula was obtained from Corine Land Cover 2000 at a 100 × 100 m resolution. The percentage of coverage in each category within each 100 km^2^ UTM cell was calculated and used as 15 explanatory variables for the analyses (see Table 1). The heterogeneity of land use types within each UTM was summarized by the Shannon diversity index and included as another explanatory variable.

Finally, spatial variables were used to verify the existence of spatial gradients. They were defined as the central latitude and longitude of each UTM cell and were included in the analyses as a third degree polynomial (Legendre and Legendre 1998). The nine terms of the spatial polynomial can help to incorporate effects of other historical, biotic or environmental variables not otherwise taken into consideration (Legendre and Legendre 1998). Latitude and longitude were standardized (mean = 0, and standard deviation =1) as were topographic, climatic, and vegetation productivity variables in order to eliminate their measurement scale effects.

**Table 1.**
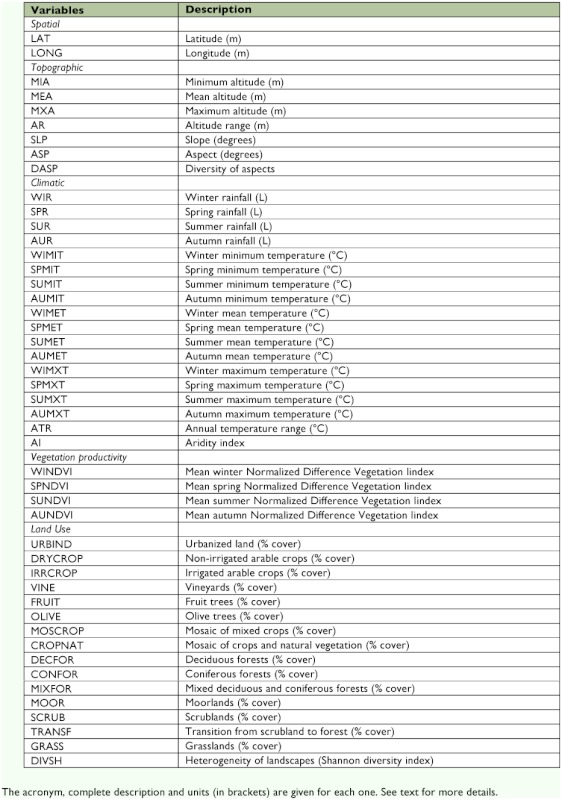
List of explanatory variables used in analyses.

### Statistical analyses

Multiple regression models implemented in the General Regression Models module of STATISTICA (StatSoft 2001) were conducted to determine the relationship between response (appearance date of *A. mellifera* and *P. rapae*) and explanatory (spatial, topographic, climatic, vegetation productivity and land use) variables. Models were built in three sequential steps. First, the relationship between insect appearance and each explanatory variable was explored one-by-one. For each predictor, linear, quadratic or cubic relationships were sought. The functions whose terms were statistically significant (p < 0.05) were selected. Only those environmental variables that were significantly related to appearance dates were included in further analyses. In the second step, the modelling ability of each type of explanatory variable (i.e. spatial, topographic, climatic, vegetation productivity and land uses) was explored by including significant variables belonging to the same category in a single regression model. A procedure of backward stepwise selection was applied in all cases to obtain simplified models that included only significant variables. The model with spatial variables allowed assessment of the spatial structure of phenological data, while models with environmental variables (i.e., the rest of the predictors) constituted the rationale for a biological interpretation of the spatial structure of dates that was identified. In the third and final analytical step, the best explanatory model of appearance dates was sought. For this purpose, two different regression models were carried out. One was with environmental explanatory variables and the other included environmental and spatial variables. The nine terms of the third degree spatial polynomial included in the latter model seek to account for the potential effect of other non-considered variables that are spatially structured. A backward selection procedure was applied in both models to include only significant (p < 0.05) predictors. Predicted scores of the best final regression model were mapped and examined.

Explanatory variables were correlated to each other (i.e., multicollinearity) due to the cofluctuation imposed by spatial and environmental gradients of the Iberian Peninsula (e.g., northern and high altitude regions have cooler and moister climates). This fact hinders the estimation of the true relevance of predictors (Quinn and Keough 2002). A hierarchical partitioning of variance procedure (Birks 1996; MacNally 2000, 2002) was implemented to determine the relative importance of each type of explanatory variable. The relative importance can be estimated as the average effect of including each type of variable in all possible models built with the remaining types of variable. Therefore, 2^*k*^ functions must be constructed for *k* types of explanatory variables. In our case, *k* = 5 (spatial, topographic, climatic, vegetation productivity and land uses) and thus 2^*k*^ = 32 different models.

Residuals from all multiple regression models were examined to check for spatial autocorrelation. If residuals are spatially autocorrelated, one or several important spatially structured explanatory variables are left out of the models (Cliff and Ord 1981; Legendre and Legendre 1998). Moran's *I* autocorrelation coefficient with a Bonferroni-corrected significance level (Sawada 1999) was calculated against ten classes separated by a lag distance of 60 km (from 60 to 600 km).

## Results

### Apis mellifera

**Figure 2.**
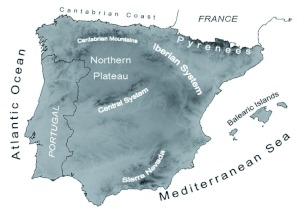
Topographical map of the Iberian Peninsula. Darkness is proportional to the elevation. Main geographical features cited in the text are also shown. Solid black lines indicate political borders. High quality figures are available online.

Many localities with an early phenology (especially those with very early appearance dates, i.e., prior to mid-February) were located in the southern and coastal areas of Spain (Figure 1a). In contrast, late sites for bee phenology occur mainly in central Iberia (i.e. Northern Plateau) and some mountainous regions (e.g., Iberian System; see Figure 2). Nevertheless, this row pattern is blurred by variability in the smaller scale. There were large differences between neighbouring UTMs. This could explain why the assessment of the spatial structure of data (i.e., the spatial model) showed a moderate explanatory ability (Table 2). The only variable included in the spatial model was the cubic function of the latitude. In agreement both with the prediction and the visual inspection of raw data, the spring appearance of *A. mellifera* is later in northern areas (*c*. 3 days·degree of latitude^-1^).

**Figure 3.**
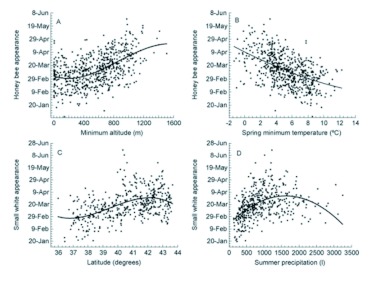
Scatterplots of insect appearance against the best environmentally-related variables. Each point represents the average date of first appearance of *Apis mellifera* or *Pieris rapae* in each sampled UTM and its corresponding value of a selected environmental variable (see x-axis). *A. mellifera* appears later in those UTM cells with a higher minimum altitude (a) and earlier in those with warmer spring temperatures (b). The first *P. rapae are* sighted in the southern (c) and driest UTMs (d). Solid lines depict the best polynomial fitted model. High quality figures are available online.

The topographical model included only the minimum altitude, the cubic function of which was strongly fitted to appearance dates (Table 2). *A. mellifera* appears later in those localities that are at higher elevations (Figure 3a). The effect was especially marked in those UTMs with a minimum altitude above 300 m.

**Table 2.**
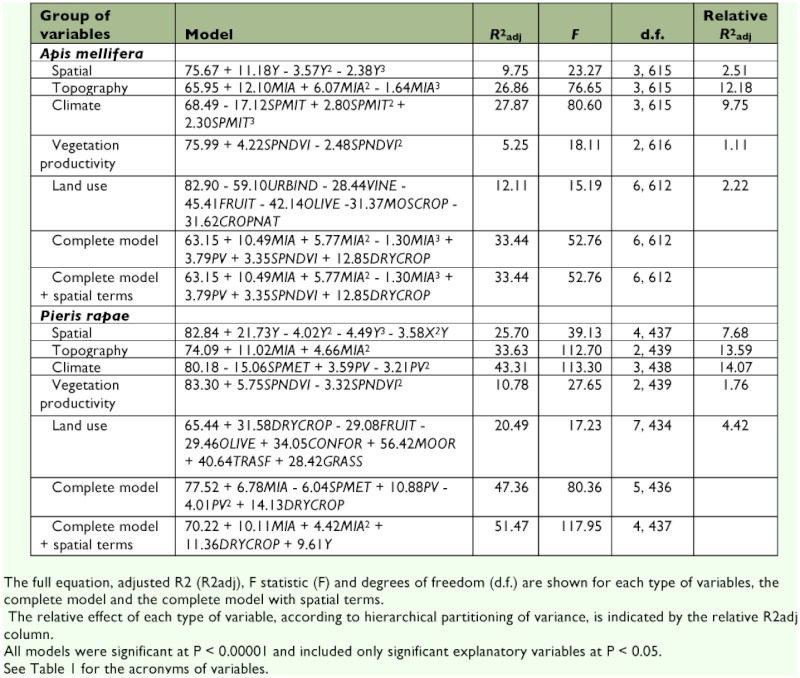
Best multiple regression models for appearance dates of the honey bee and small white.

The climatic model was the best environmental model (Table 2), although just one variable — the spring minimum temperature — was included. The paucity of variables in this model is remarkable because all climate variables (except autumn rainfall, see Table 1) were each significantly related to *A. mellifera* appearance dates. UTMs with the earliest bee appearance were located in those areas with the warmest springs (Figure 3b).

The model obtained with vegetation productivity variables was the least explanatory (Table 2). The quadratic function of the spring NDVI was able to capture just 5.55% of the variability in appearance dates. The appearance of *A. mellifera* was earlier in regions that are less productive during the spring.

Up to six variables were significantly included in the land-use model, although they accounted for a low proportion of the variability in appearance dates overall (Table 2). The negative sign of all variables means that *A. mellifera* were recorded earlier in the UTMs with large coverage of urbanized (i.e. humanized) areas, vineyards, fruit trees, olive groves, croplands and wild vegetation.

The average relative explanatory capacity of each variable was notably lower than that obtained in previous models (Table 2). This could have been because of the high degree of collinearity among the explanatory variables. Nevertheless, the types of explanatory variables that showed the best fitted models also accounted for greater fractions of variability themselves. Interestingly, the variance hierarchical partitioning procedure revealed that the topographic model explained, on average, 2.4% more of the variability than the climatic model (Wilcoxon Matched Pairs test: Z_16_ = 3.154, p = 0.002).

**Figure 4.**
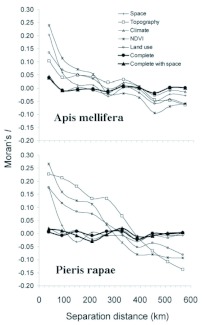
Spatial autocorrelation of model residuals. The isotropic correlogram represents the variation in the scores of Moran's I spatial autocorrelation statistic with the increasing separation distance between UTM cells, using a lag distance of 60 km and an active lag of 600 km. Spatial autocorrelation was absent at any distance in complete models (thick lines). High quality figures are available online.

The complete environmental model included four variables and captured a bit more variability in appearance dates than the topographic or climatic models (Table 2). Minimum altitude and spring vegetation productivity had the same effect (i.e. sign) that was described above. The cubic function of the minimum altitude was the most relevant predictor of this model (partial *R*^2^ = 22.81%). One climatic and one land use variable that were not included in their respective models were included in the final model. Although they did not show the best explanatory capacity within their groups of variables, they likely worked on a different part of the variability of dates. Consequently, summer precipitation and cover of dry farming were not overridden by altitude effects. According to the positive sign of summer precipitation and percentage of dry farming, localities with an early appearance of *A. mellifera* were related to regions with little precipitation during the summer and with a low proportion of dry farming. Spatial terms were not included in the complete model after the backward selection procedure, and, consequently, the model remained equal. Hence, no spatial structure remained in the phenological data after modelling with these environmental variables. This agrees with the absence of spatial autocorrelation in residuals of the complete model (Figure 4).

**Figure 5.**
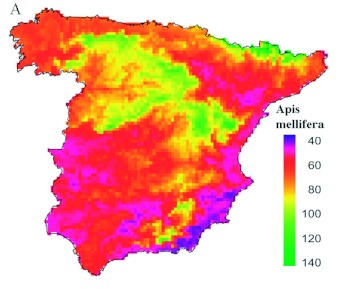
Maps of the predicted appearance dates for *A. mellifera* in Spain according to the best complete models. Scale color bar in Julian days (1 = 1 January).High quality figures are available online.

When predicted appearance dates from the complete model were mapped, the pattern of bee appearances in Spain was clearer than that offered by raw data (Figure 5). *A. mellifera* were observed for the first time on the southeastern Mediterranean coast of Spain. Other markedly early regions that were predicted included the southern regions and the Mediterranean coastal regions. Immediately later, *A. mellifera* appeared during the first 10 days of March in most of Spain. Finally, the late appearance of *A. mellifera* (from April 1st onwards) was expected in some central (Northern Plateau) and mountainous (Iberian System, Sierra Nevada or Pyrenees) regions of the Iberian Peninsula as a consequence of the high altitude.

### Pieris rapae

**Figure 6.**
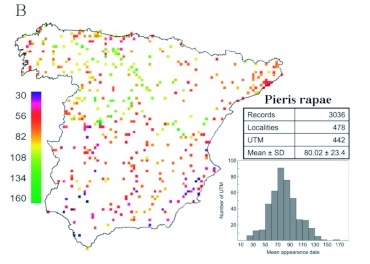
Maps of the distribution of phenological records of the appearance dates of *Pieris rapae.* Each square represents a UTM with phenological records, and its colour depicts the mean date. Scale color bar in Julian days (1 = 1 January). The total number of records, localities, and UTM together with the mean value and the standard deviation (SD) of all records are also specified. A histogram with the distribution of mean dates per UTM is also shown (scale of x-axis in Julian days). High quality figures are available online.

A visual examination of raw data offered more evident spatial patterns for *P. rapae* appearances than observed for *A. mellifera* (Figure 6). Regarding the appearance of these insects, Spain can be unambiguously divided into two regions: one early area in the southern half of the Iberian and a late area in the northern half. Coastal areas both along the Mediterranean and along the Cantabrian seas also showed early appearance dates. Such patterns support a spatially well-structured *P. rapae* phenology. Indeed, all regression models for this species were better fitted (i.e., had a higher *R*^2^) than those obtained for *A. mellifera* (Table 2). This is especially striking because the explanatory variables included in the models were the same in most cases. Therefore, differences in the models' fitting between *A. mellifera* and *P. rapae* are likely not due to a different composition of the included explanatory variables. The spatial model showed a strong cubic latitudinal gradient (partial *R*^2^ for latitude = 21.82%). The appearance of *P. rapae* was delayed almost one month from the southernmost point of Spain to the Cantabrian Mountains (Figure 3c). In the small latitudinal range from the Cantabrian Mountains to the Cantabrian coast, appearance dates tended to be earlier. Such a gradient was likely a result of the milder conditions due to the low altitude and to the proximity to the Atlantic Ocean.

The minimum altitude also played an important role in the spatial variability of *P. rapae* phenology (Table 2). Those UTMs with higher minimum altitudes showed later appearance dates. The effect of minimum altitude was especially pronounced at higher altitudes, as was stressed by the quadratic relationship (see Figure 3a).

The climatic model was also the best model for *P. rapae*, although in this case, the explanatory capacity was markedly higher than the topographic model (Table 2). Temperature was the best explanatory variable. The linear relationship of appearance dates against mean spring temperatures accounted for 41.18% of the variability of *P. rapae* phenology. *P. rapae* is recorded earlier in areas that are warmer during the spring. The quadratic function of the summer rainfall was also included in the climatic model. This variable notably modeled appearance dates (*R*^2^ = 26.2%; Figure 3d). The earliest appearances were linked to the driest areas. The early appearance in the moistest UTMs is linked to the latitudinal gradient. The moistest areas of the Iberian Peninsula are located on the Cantabrian coast, which has unusually mild conditions due to its proximity to the sea. The strong explanatory capacity of temperature and precipitation models did not add up as a result of the strong collinearity of both variables. Consequently, the full climatic model fit slightly more than previous models with a single climatic variable (Table 2). Nevertheless, the mean spring temperature was the most important variable in the climatic model (partial *R*^2^ = 38.50%).

Vegetation productivity variables produced the worst explanatory model (Table 2). Spring patterns of productivity were related to appearance dates in a quadratic form. Early dates were linked to regions with low NDVI values during the spring.

The land use model included a large number of variables (Table 2) and showed a moderate explanatory capacity of *P. rapae* phenology (Table 2). The first butterflies are recorded in areas with large coverage of olive groves and fruit trees, but with a low coverage of dry farming, coniferous forests, moorlands and transitional areas from shrublands to forests and grasslands. This result concurs with the spatial meaning of previous models. Fruit trees and olive groves are located mainly in southern Spain and on the Mediterranean coast. However, dry farming dominates landscapes from the Northern Plateau, and coniferous forests, moorlands and grasslands are typical for mountainous regions with high elevations, low temperatures and moist climates.

The average percentage of variability that accounted for each type of variable (see relative *R*^2^_adj_ in Table 2) was much lower, considering the high degree of collinearity among explanatory variables. Nevertheless, the relevance order was maintained. Topographic and climatic variables accounted for greater fractions of variability by themselves. Interestingly, true differences in the explanatory capacity of topographical and climatic variables were not too large, according to the relative *R*^2^ values. On average, vegetation productivity and land use captured a negligible part of the variability.

The best complete model was built with climatic variables, minimum altitude and the coverage of dry farming (Table 2). The addition of minimum altitude and coverage of dry farming to climatic variables minimally increased the explanatory capacity previously achieved by the climatic model. The inclusion of spatial terms improved explanatory capacity of the model. This was due to the replacement of climatic variables with latitude. Residuals from both complete models (with and without spatial variables) did not show significant autocorrelation scores at any lag distance (Figure 4). Therefore, all spatially structured variations were included in *P. rapae* models.

**Figure 7.**
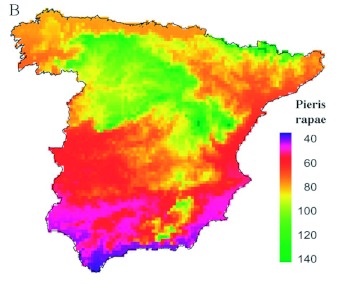
Maps of the predicted appearance dates for *P*. *rapae* in Spain according to the best complete models. Scale color bar in Julian days (1 = 1 January). High quality figures are available online.

Predicted date scores from the best complete model showed plainly differentiated phenological regions (Figure 5b). The earliest appearances of *P. rapae* were in the southern coast of Spain on both the Atlantic and the Mediterranean sides. However, the latest appearance dates were in the inner regions of the Northern Plateau and the Iberian System, as well as in the mountainous regions of the Pyrenees and Sierra Nevada. Intermediate regions also show a clear latitudinal gradient with later appearance dates in the Cantabrian coast and the Ebro valley than the dates in the Levant coast and southwestern Spain.

## Discussion

Spatial patterns of *A. mellifera* and *P. rapae* appearance dates were strongly related to environmental gradients, especially those related to temperature. In fact, temperature effects were both direct (see Figure 3) and indirect. The indirect effect was through other environmental and geographical gradients, such as altitude (see Fig. 3) and latitude (see Figure 3c). This strong effect of temperature on insect phenology was already demonstrated for the interannual variability of spring appearance phenology of the same species in the same area (Gordo and Sanz 2006b). Therefore, temperature was the most important factor in the year to year fluctuations in appearance dates (Sparks and Yates 1997; Roy and Sparks 2000; Dell et al. 2005; Gordo and Sanz 2005), but was also the most important factor in the site to site variability. Such strong effects of temperature were expected because insects are poikilotherms, and, thus, their activity, development, and vital cycles are heavily influenced by weather.

The best complete models showed a good ability to model regional differences in phenological dates (especially in the case of *P. rapae*). In a previous study (Gordo and Sanz 2006b), it was found that the appearance of *A. mellifera* and *P. rapae* had significant latitudinal, longitudinal, and altitudinal gradients. The results for latitude and altitude were confirmed here. Significant effects of longitude were not found, but as was pointed out by Gordo and Sanz (2006b), the effect of this variable was negligible. In any case, the explanatory capacity of the models in the present study performed better than previous models suggested by Gordo and Sanz (2006b), which were focused on temporal (i.e., interannual) variability rather than on spatial (i.e., intra-annual) variability. They did not further explore the biological meaning of spatial gradients that they found.

One of the most interesting peculiarities of these models (see Table 2) is the small number of explanatory variables included in them, despite the plethora of predictors employed (see Table 1). For example, just three variables were necessary to explain more than half of the spatial variability of *P. rapae.* Most explanatory variables were ruled out during the stepwise selection due to their redundancy. This strong collinearity was expected because all variables belong to a common spatial scenario (i.e. the Iberian Peninsula), and, consequently, all of them depicted the same environmental gradients. Furthermore, residuals from complete models did not show spatial autocorrelation, and thus there was no remaining spatial variability in the phenological data at this working scale. Therefore, the explanatory capacity of complete models could not be improved by adding other types of variables. In any case, the included variables and their effects in all types of models (i.e. climatic, topographical, etc) pointed toward the same spatial gradients: insects appear later in cooler areas, which correspond to high altitude and northern regions that receive higher amounts of summer precipitation and are dominated by dry farming. The effect of dry farming is probably due to the influence of the Northern Plateau region (see Figure 2). This is a high altitude region with a marked continental climate in the northern half of Iberia, where land use is dominated by extensive cereal areas.

Both species have very similar appearance dates (*A. mellifera* appears just one week earlier than *P. rapae*), and thus both are under very similar ecological influences, such as photoperiod and spring temperatures. This opens the possibility of determining to what extent spatial variability is under common environmental constraints or is dependent on species' biology peculiarities (Gordo et al. 2008). Both species showed models composed of similar predictors, but the explanatory capacity and predicted maps of the complete models (see Figures 5 and 7) were different. Differences in explanatory capacity could be due to differences in the strategies used by each species to survive during the winter. *A. mellifera* hibernates as adults, while *P. rapae* hibernates as quiescent pupae. *A. mellifera* may react faster than *P. rapae* to increasing temperatures during the spring. *A. mellifera* only must stop its lethargy, while *P. rapae* must complete part of its development (Gordo and Sanz 2006b). Consequently, *A. mellifera* may be more influenced by climatic conditions on a microscale level (e.g., beehive located in a southern exposure), which would be unable to be modelled at this work resolution and extent. Such local environmental peculiarities would add white noise to the macroscale gradients of the Iberian Peninsula. However, *P. rapae* requires the constancy of raised temperatures to complete its development. As a consequence, spatial variability of *P. rapae* appearance phenology would fit much better with the spatial gradients of temperature during the spring. Nevertheless, almost half of the variability among localities remains to be explained for *P. rapae,* which could also be evidence of the effect of microclimatic conditions at each study site.

Land-use and the NDVI explanatory variables showed a poor ability to model insect appearance (see relative *R*^2^_adj_ values in Table 2). In fact, landscape composition and vegetation productivity were not associated with any strong phenological gradient shown by bees or butterflies in the Iberian Peninsula. These results agree with the main findings of Cocu et al. (2005) for aphid phenology. The main predictor of aphid flying dates was climate (see also Harrington et al. 2007), while differences in land use had a negligible effect. One possible reason for the poor modelling ability of land use could be purely methodological. The present distribution of land use was used, while phenological data have been recorded during five decades. Land use has changed during the last five decades (Pavón et al. 2003; Calvo-Iglesias et al. 2006). Thus, the present landscape may be not fully representative of the past landscape structure, when part of the data set was recorded. Nonetheless, this possibility can be ruled out because landscape changes occur on a rather small spatial scale without any coherent spatial pattern (e.g., afforestation is a generalized phenomenon). The most plausible hypothesis for the lack of land use effect is the working extent. The largest differences in appearance dates were imposed by large scale trends (e.g., south-north), which are beyond the local or regional scale where land use patterns exist. In the case of the NDVI, the poor modelling performance suggests that the proposed hypothetical link among vegetation productivity, insect abundance, and phenology could be ruled out.

Among climate variables, summer rainfall had a noticeable effect (Figure 3d). This merits interest because insect appearance occurs during the spring. The “advanced” effect of this variable has been demonstrated for other spring phenological events, such as the arrival of migratory bird species (Gordo et al. 2007a, b). This generalized effect of summer rainfall over a wide range of species suggests that organisms arrange their life cycles according to constraints imposed by aestival conditions in the Mediterranean. Alternatively, the summer rainfall effect may be due to the strong collinearity between summer rainfall and spring temperature patterns (*r* = -0.72 for the 6,063 UTM cells of Spain). Those areas with the warmest spring temperatures became, several months later, those areas with the driest summer. High temperatures during the spring also encourage an early phenology. This would allow all spring processes (e.g., reproduction) to finish prior to the arrival of harsh summer conditions. Therefore, the advance of phenology in response to scarce summer rainfalls could be mediated by high temperatures during the spring. Unfortunately, the correlative approach in this study does not allow for differentiating the true acting mechanism of summer rainfalls over phenology.

Comparing the spatial patterns of insect appearance with the arrival patterns of migratory birds revealed that the environmental determinants in each case are different. Insect phenology is driven by climatic gradients, while the arrival of birds is strongly influenced by geographical configuration (such as mountains, valleys, etc.), which shapes the optimum progression routes through the Iberian Peninsula (Gordo et al. 2007a, b, 2008). As a consequence of these differences, insect appearance patterns (see Figures 5 and 7) depict a different picture with regard to migratory birds (Gordo et al. 2007a, b, 2008). In fact, the above mentioned differences between *A. mellifera* and *P. rapae* patterns become of minor relevance when insect patterns are compared to bird patterns. Such differences are paramount because most migratory birds rely on insects to feed. The earliest appearances of insects occur in the southern coast of Spain or even in the southeastern coast, as in the case of *A. mellifera* (see Figure 5). However, Gordo et al. (2007a, b, 2008) found that the earliest arrivals of birds occur in southwestern Spain, while the southeastern corner of the Iberian Peninsula showed a delayed arrival due to the geographical difficulties in being reached from the Straits of Gibraltar. Therefore, the phenological scenario found by migratory birds in different regions of Spain is not comparable. For instance, birds arrive later to southeastern Spain than to southwestern Spain, while insect phenology shows similar dates in both regions. Therefore, birds breeding in southeastern Spain found a more advanced insect phenological scenario. Similarly, a relatively late arrival of birds with regard to insect appearance also occurs at most coastal sites. Hence, spatial patterns found for insects support the geographical constraint hypothesis proposed by Gordo et al. (2007a) for migratory birds. Unfortunately, this reasoning has two weaknesses: 1) spatial patterns were explored for only two insect species (*A. mellifera* and *P. rapae*), which are not necessarily representative of all insects, and 2) insectivorous migratory bird species may rely on insect prey other than bees and butterflies. Nevertheless, the strong dependence of both studied insect species on temperature gradients, which has been found in other insect taxa (e.g., aphids: Cocu et al. 2005, Harrington et al. 2007; spittlebugs: Fielding et al. 1999), suggests that the spatial patterns found here are probably representative of the spatial gradients in the phenology of other insect communities. When organisms are altering their life cycles in response to climate change, it is of paramount importance to know the extent that species' phenological responses will maintain the phenological matching with their environment (Visser and Both 2005). However, the potential constraint imposed by the differential role of determining environmental factors in the spatial variability in each species has been skipped.

The Spanish phenological network was created with an applied intent. In fact, *A. mellifera* and *P. rapae* were selected by their relevance for agriculture. The results also offer information of applied interest. For example, more severe damage to cabbage crops by *P. rapae* larvae can be predicted in the southern and warmer areas of Spain due to an earlier initiation of their life-cycle (up to three months). Consequently, individuals may complete more cycles (i.e., multivoltinism) there. Unfortunately, no data exists concerning damage caused by *P. rapae* to evaluate this hypothesis. In the same way, *A. mellifera* resume their activity with a noteworthy spatial variability, which should be taken into account for entomophilous crops. A correct selection of plant species or varieties with a suited phenology would be necessary to ensure fertilization of flowers by this pollinator species and an optimum crop yield.

## Abbreviations

AI: aridity index;
NDVI: normalized difference vegetation index;
UTM: universal transverse mercator
